# Internal dosimetry in F-18 FDG PET examinations based on long-time-measured organ activities using total-body PET/CT: does it make any difference from a short-time measurement?

**DOI:** 10.1186/s40658-021-00395-2

**Published:** 2021-07-15

**Authors:** Pengcheng Hu, Xin Lin, Weihai Zhuo, Hui Tan, Tianwu Xie, Guobing Liu, Shuguang Chen, Xin Chen, Haojun Yu, Yiqiu Zhang, Hongcheng Shi, Haikuan Liu

**Affiliations:** 1 Department of Nuclear Medicine, Zhongshan Hospital, Fudan University, 1069 Xietu Road, Shanghai, 200032 China; 2 Institute of Radiation Medicine, Fudan University, 2094 Xietu Road, Shanghai, 200032 China

**Keywords:** Time-activity curve, Cumulated activity, Organ absorbed dose, Dynamic bladder wall absorbed dose, Effective dose, Total-body positron emission tomography

## Abstract

**Purpose:**

A 2-m axial field-of-view, total-body PET/CT scanner (uEXPLORER) has been recently developed to provide total-body coverage and ultra-high sensitivity, which together, enables opportunities for in vivo time-activity curve (TAC) measurement of all investigated organs simultaneously with high temporal resolution. This study aims at quantifying the cumulated activity and patient dose of 2-[F-18]fluoro-2-deoxy-D-glucose (F-18 FDG ) imaging by using delayed time-activity curves (TACs), measured out to 8-h post-injection, for different organs so that the comparison between quantifying approaches using short-time method (up to 75 min post-injection) or long-time method (up to 8 h post-injection) could be performed.

**Methods:**

Organ TACs of 10 healthy volunteers were collected using total-body PET/CT in 4 periods after the intravenous injection of F-18 FDG. The 8-h post-injection TACs of 6 source organs were fitted using a spline method (based on Origin (version 8.1)). To compare with cumulated activity estimated from spline-fitted curves, the cumulated activity estimated from multi-exponential curve was also calculated. Exponential curve was fitted with shorter series of data consistent with clinical procedure and previous dosimetry works. An 8-h dynamic bladder wall dose model considering 2 voiding were employed to illustrate the differences in bladder wall dose caused by the different measurement durations. Organ absorbed doses were further estimated using Medical Internal Radiation Dose (MIRD) method and voxel phantoms.

**Results:**

A short-time measurement could lead to significant bias in estimated cumulated activity for liver compared with long-time-measured spline fitted method, and the differences of cumulated activity were 18.38% on average. For the myocardium, the estimated cumulated activity difference was not statistically significant due to large variation in metabolism among individuals. The average residence time differences of brain, heart, kidney, liver, and lungs were 8.38%, 15.13%, 25.02%, 23.94%, and 16.50% between short-time and long-time methods. Regarding effective dose, the maximum differences of residence time between long-time-measured spline fitted curve and short-time-measured multi-exponential fitted curve was 9.93%. When using spline method, the bladder revealed the most difference in the effective dose among all the investigated organs with a bias up to 21.18%. The bladder wall dose calculated using a long-time dynamic model was 13.79% larger than the two-voiding dynamic model, and at least 50.17% lower than previous studies based on fixed bladder content volume.

**Conclusions:**

Long-time measurement of multi-organ TACs with high temporal resolution enabled by a total-body PET/CT demonstrated that the clinical procedure with 20 min PET scan at 1 h after injection could be used for retrospective dosimetry analysis in most organs. As the bladder content contributed the most to the effective dose, a long-time dynamic model was recommended for the bladder wall dose estimation.

**Supplementary Information:**

The online version contains supplementary material available at 10.1186/s40658-021-00395-2.

## Introduction

Positron emission tomography (PET) provides unique information about the molecular and metabolic changes associated with disease [1]. Internal dosimetry is not only important in the radiation protection but also in optimizing injection dose, improving image quality, and streamlining clinical workflow.

Dynamic PET can acquire the spatiotemporal distribution of radiotracers in vivo, which could be used for organ TAC measurement and the absorbed dose calculation after the intravenous administration of F-18 FDG [2–8]. Based on previous TAC data [2–6], ICRP 106 reported a series of biokinetic data and administered activity to dose conversion factors [9]. Thereafter, multiple studies applied the recommended F-18 FDG biokinetic data and the administered conversion factors to estimate patient internal radiation dose [10–14]. Recently, voxel S-value-based (VSV) method has been shown as a more accurate means for patient-specific dosimetry [15], which also requires an accurate measurement of cumulated activity at voxel level.

However, the TAC data used for cumulated activity calculations and dose estimation in previous studies was mostly collected either using traditional PET scanners within relatively short duration. Studies using traditional PET scanners can only measure activity of limited number of organs, while assuming other organs of the body follow uniform radioactivity distribution [2, 3, 5–8]. Dosimetry works using whole-body dynamic PET can only acquire limited temporal frames within 80 min [3, 4] due to insufficient PET system sensitivity and coverage, and hence, multi-exponential functions with different assumptions were employed to fit the TACs of a short duration [3–8]. One of these assumptions is that the activity only decreased by physical decay after the last frame, while others includes the biological clearance into the model. The newly developed high sensitivity dynamic total-body scanner with an axial field of view of 194 cm and a transaxial field of view of 68.6 cm is able to perform a head-to-toe dynamic PET scanning simultaneously [16, 17], which allows for measurement of TAC for all organs simultaneously with high temporal resolution. The accurately measured TACs could be used to clarify the differences in cumulated activity caused by the duration of measurement and various assumptions.

Furthermore, all previous studies assumed that the bladder content volume remained constant, which could attribute significant differences in absorbed dose estimation [17, 18]. Wu et al. and Dowd et al. built a dynamic bladder wall radiation dose model where the bladder wall radiation dose caused by the content was a function of dynamic bladder volume and time activity of the bladder content. However, this model assumed that urine excretion following the first or second voiding was negligible in calculation of the absorbed dose of the bladder wall because of the short half-life of F-18 FDG [18–20]. A two voiding model and 8 h model were employed in this study to investigate the potential difference caused by a short-time measurement of urine and bladder activity.

In this study, total-body dynamic PET scan out to 8 h after the intravenous injection of FDG was carried out using the newly developed scanner to obtain a quantification of the radioactivity of multiple organs. A comparison of cumulated activity, bladder wall dose, and effective dose estimated using classic short-time measurement (up to 75 min post-inject) and long-term measurement (up to 8 h post-injection) was conducted.

## Materials and methods

### Subjects

This study was approved by the Medical Ethics Committee of Zhongshan Hospital, Fudan University, and inform consented was obtained. Ten healthy volunteers (7 men, 3 women; mean age, 55.7 ±9.5 years, mean ±standard deviation) without claustrophobia participated in this study. The average height and weight of the male subjects were 166.9 ±3.7 cm (mean ±standard deviation) and 63.6 ± 8.6 kg, respectively, and for female subjects the average height and weight were 153.7±7.5 cm and 56.2±7.4 kg. Before performing the PET/CT imaging, all subjects were asked to refrain from any medication and to fast at least for 6 h, and their blood glucose was measured by blood sampling.

### Total-body PET scanning protocol

**Fig. 1:**
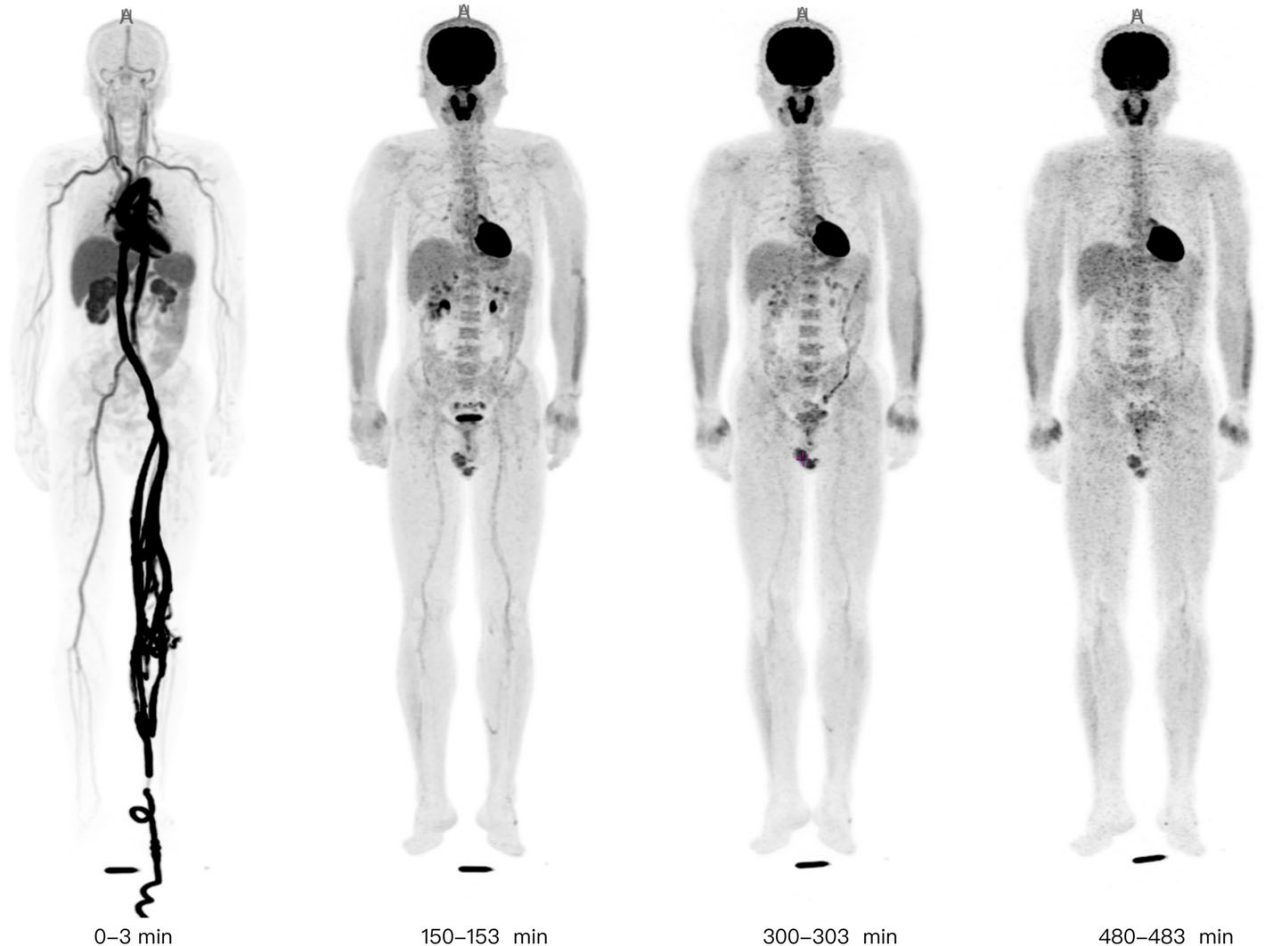
Reconstructed dynamic PET images (Maximum-Intensity Projection) of early 3 min, 150~153 min, 300~303 min, 480~483 min (with calibration source close to the feet)

A 75-min duration scan was performed immediately after an intravenous injection of F-18 FDG via a vein near the ankle; a dosing regimen of 1.85 MBq/kg was used. The list-mode PET data was acquired on a 194-cm long axial FOV (an axial acceptance angle of ∼± 57°) total-body PET/CT (uEXPLORER, United Imaging Healthcare, Shanghai, China) [21]. Low-dose CT scans were obtained for attenuation correction and all corrections were applied to the reconstructed images. A high temporal resolution dynamic reconstruction protocol was used for the initial 75-min duration scan, with the dataset divided into 60 frames: 36 × 5 s, 24 × 180 s. Another three PET scans of 15-min duration were conducted for each subject approximately at 150, 300, and 480 min after the intravenous injection; these three delayed scans used a dynamic reconstruction protocol of 5 frames: 5 × 180 s. Example reconstructed dynamic PET images in 4 periods for a single subject is shown in Fig. 1. All PET images were reconstructed using ordered subset expectation maximization (OSEM) algorithm with the following parameters: TOF and PSF modeling, 2 iterations and 20 subsets, matrix 192 × 192, slice thickness 2.89 mm, FOV 600 mm (pixel size 3.125 × 3.125 × 2.89 mm^3^) with a Gaussian post-filter (3 mm), and attenuation and scatter correction. All images were transferred to a commercial medical image processing workstation (uWS-MI, United Imaging Healthcare) for the image evaluation and quantitative analysis.

### Time-activity curve measurement

The radioactivity in major organs was obtained from reconstructed dynamic PET to low-dose CT fused images by manual contouring based on anatomical CT images. The volume of interest (VOI) contouring method was based on the study by Deloar H.M. et al. [4] The TACs of 6 organs in each subject, including brain, heart (left ventricle myocardium only), kidneys, liver, lungs, and urinary bladder, were obtained. VOI analysis was carried out with a built-in PET image analysis software uWS-MI. To ensure the accuracy of the activity, a radioactive standard source of 1 ml tube of F-18 FDG solution was used to calibrate the activity. The activity measured both by PET reconstruction and radiation dosimeter was recorded to calibrate the activity measured by PET. In that way, an extra calibration source was placed next to the subject during acquisition to confirm the calibration of the system besides regular quality assurance.

### Cumulated activity calculations

Because the decay and most of the metabolic processes are exponential, multi-exponential function is widely adopted to fit TAC data in PET images and we followed the previously reported models for multi-exponential fitting of the short-term data in the present work [2–4, 7]. Recently, the time-activity curves represented by a sum of temporal basis functions, for instance, B-splines, have been proposed in parametric estimation in dynamic PET [22–24]. For spline method, the area under TAC was calculated as follows:

1 $$ \overset{\sim }{A_s}={\int}_0^TA(t)\  dt+{\int}_T^{\infty }{A}_f{e}^{-\lambda t} dt $$

where $\overset{\sim }{A_s}$ is the cumulated activity, *A*(t) is the spline fitted activity curve, *A*_*f*_ is the activity of the organ at the end of the last PET scan when the emission scan was assumed to begin to decrease only by radiative decay, and *λ* is the decay constant.

The spline fitting and mathematical integration process was done using Origin (version 8.1).

The time activity curve for urine remained in the urinary bladder; *A*_*u*_(*t*) was fitted with the sum of two exponentially decaying functions and one constant as follows:

2 $$ {A}_u(t)={e}^{-\lambda t}\times \left({A}_1{e}^{-{k}_1t}+{A}_2{e}^{-{k}_2t}+C\right)-{\sum}_{i=1}^{\infty}\varepsilon \left(t-{T}_i\right){A}_{ur}(t) $$

where *A*_1_ and *A*_2_ are the intercepts, *k*_1_, *k*_2_ are biological elimination constants, *ε*(*t*) is Heaviside function, *T*_i_ is the *i*th urinary voiding time, and *A*_*ur*_(*t*) is the activity of urine excreted out of the body. *A*_*ur*_(*t*) was measured by collecting the urine of subjects after the injection, which used a radiation dosimeter (well counter) to measure the activity.

The time-activity curves were fitted as described in previous studies [4], and residence time was derived to compare the spline method with the multi-exponential method.

To fit the equation (2), 12 frames of the organ activity data were selected from 0 to 60 min out of the 8 h measurement: 12 × 120 s as described in previous studies [2, 3, 7], and the data at 10, 40, 70 min: 3 × 180 s were also used for comparison [4].

In our current clinical practice, a PET scan of 20-min duration at 1-h post-injection is used. To evaluate the possibility of individual dose estimation with standard clinical protocols, 5 × 180 s frames of data from 57 to 75 min was used to estimate the cumulated activity, and this was compared with the estimation using the long-time, delayed scanning protocol.

### Bladder wall absorbed dose calculation

Previous studies have shown the S value brings the most uncertainty in internal dose calculation of bladder wall absorbed dose among all the organs [16, 17]. In this study, the absorbed dose of bladder wall that was caused by other organs was calculated using S-value obtained from Monte-Carlo simulation. The absorbed dose for the bladder content irradiating the bladder wall was calculated using the dynamic model, which is a function of volume and activity of the bladder content. A dynamic absorbed dose calculation method was based on the assumption that the bladder wall is primarily contributed by two parts of irradiation: the gamma photons and positrons [18, 19, 25, 26]. The average bladder wall dose per unit administered activity was described as,

5 $$ \frac{D_s}{A_0}=\frac{1}{A_0}\int \left[{D}_{\gamma }(i)+{D}_{\beta }(i)\right] dt=\frac{1}{A_0}\int \left[\frac{\phi_{\gamma }{A}_u(t)}{{\left(36\pi \right)}^{1/3}V{(t)}^{2/3}}+\frac{\Delta _{\beta }{A}_u(t)}{2V(t)}\right] dt $$

where *D*_*s*_ is the absorbed dose contributed from urine content to the bladder wall, *D*_*γ*_ is the absorbed dose contributed gamma photons, *D*_*β*_ is the absorbed dose contributed by positrons, *ϕ*_*γ*_ is the dose conversion factor from the contribution of gamma photons (*ϕ*_*γ*_=404.71*cm*^2^ ∙ *μGy*/(*MBq* ∙ min)), and ∆_*β*_ is mean positron particle energy emitted per nuclear transition of the radionuclide (∆_*β*_=2.3 × 10^3^*g* ∙ *μGy*/(*MBq* ∙ min)) [20].

In this study, we assumed that the urine increases at the same rate between two successive voids:

6 $$ V(t)=\left\{\begin{array}{c}{V}_0+\int u(t) dt;0\le t<{T}_1\\ {}{V}_b\left({t}_i\right)+\int u(t) dt;{T}_{n-1}\le t<{T}_n\end{array}\right. $$

where *u*(*t*) represents urine production rate.

For dynamic model considering two voiding, absorbed dose after second voiding was assumed to be zero.

### Organ absorbed dose calculation

In order to make a comparison of changes of effective dose using different measurement methods, it is necessary to calculate the organ absorbed dose to estimate the effective dose. The calculation of the dose was made via MIRD method as follows [27, 28]:

9 $$ \frac{D_{rk}}{A_0}=\sum \frac{\overset{\sim }{A_i}}{A_0}S\left({r}_k\leftarrow {r}_h\right)=\sum {\tau}_lS\left({r}_k\leftarrow {r}_h\right) $$

where *D*_*rk*_ is the absorbed dose to a target organ, *S*(*r*_*k*_ ← *r*_*h*_) is the mean absorbed dose to a target region per unit cumulated activity in a source region, *r*_*k*_ is the target organ, *r*_*h*_ is the source organ, and *τ* is residence time.

### S value calculation

ICRP reference phantom is widely used in absorbed dose estimation [29]. However, the phantom was not based on the typical habitus found in the Chinese population. We revised the ICRP realistic reference phantom into two special phantoms for a man (height, 170 cm; weight, 60 kg) and a woman (height, 160 cm; weight, 51 kg) based on two subjects to carry out the Monte-Carlo simulation.

## Results

### Comparison of cumulated time activity

**Fig. 2:**
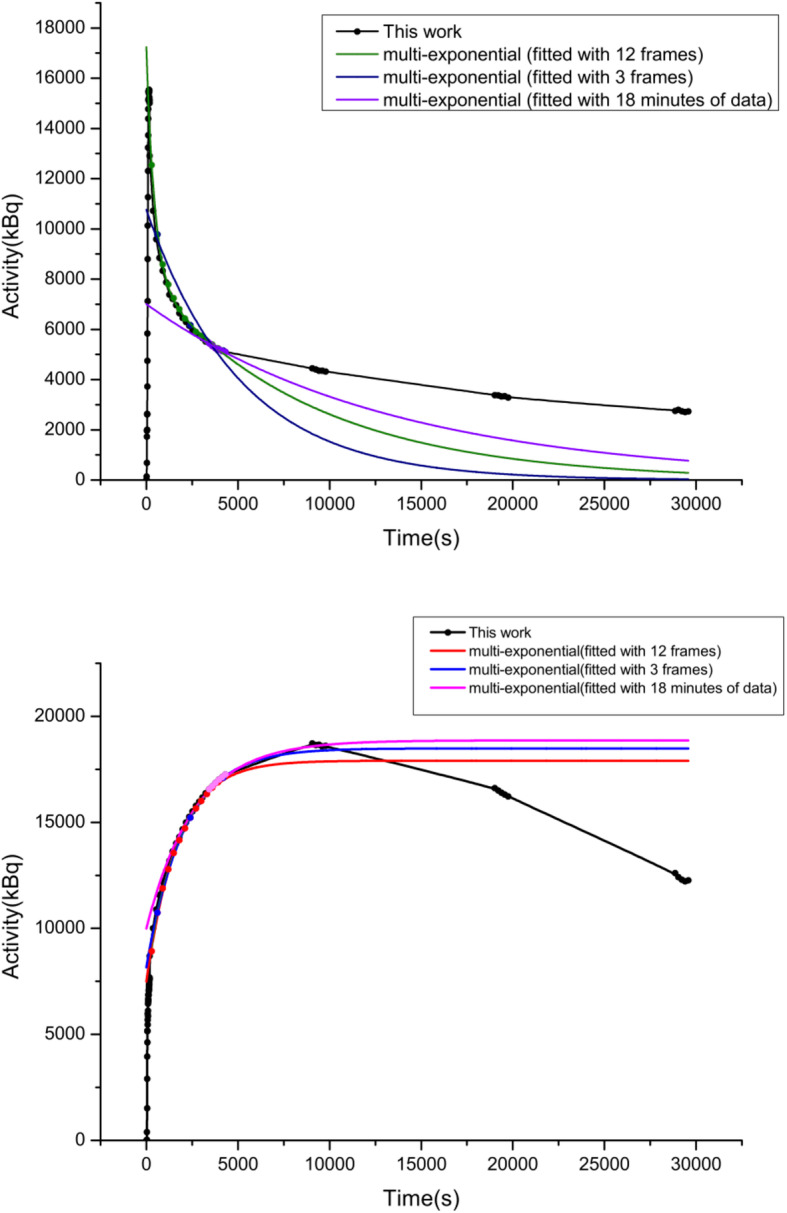
A comparison between TAC generated between multi-exponential method with 12 × 120 s, 3 × 180 s, and 18 min of data (decay corrected) and spline method (8 h, both decay corrected and not decay corrected) in liver data (up) and brain data (down) fitting in one of the studies

Spline method and multi-exponential method were applied in this study to fit the time activity curve of source organs including brain, heart, kidneys, lungs, and liver in each subject. Figure 2 shows the comparison between the multi-exponential methods and the spline method used in this study.

Figure 2 shows the differences of fitting result of TACs for spline method and multi-exponential method with 12 × 120 s and 3 × 180 s data with different assumptions as described in previous studies along with multi-exponential method with 18 min of data at 1 h after injection.

Compared with estimation using methods based on previous studies, it is obvious that the fewer data points were included, the greater bias was introduced (Table 1). The multi-exponential methods showed significant differences in residence time for the liver (18.38% on average). However, for myocardium, the differences caused by estimation methods were neglectable compared with individual variation. Assuming the activity only decreases by radioactive decay after the last scan showed significant differences in cumulated activity for the brain, liver, lungs, and kidneys (8.95%, 15.82%, 23.14%, and 22.73% on average). For organs other than liver, the multi-exponential method provided an accurate estimation of residence time; therefore, cumulated activity, based on 12 frames of data covering the first hour after injection and considering biological decay after last scan (less than 6.89% differences). The residence time of the studies where only physical decay matters after 60 to 80 min could be biased, especially when it is fitted using only three frames.

**Table 1:** Average residence time and standard deviation calculated using spline method and multi-exponential methods

Organ	Spline method	Multi-exponential method 12 frames	Multi-exponential method 12 frames (only physical decay after 60 min)	Multi-exponential method 3 frames	Multi-exponential method 3 frames (only physical decay after 70 min)	Multi-exponential with 18 min of data
**Brain**	**0.391±0.066**	0.377±0.073*	0.358±0.071**	0.380±0.067*	0.354±0.065***	0.370±0.073**
**Heart**	**0.041±0.022**	**0.044±0.021**	**0.039±0.019**	**0.040±0.023**	**0.036±0.018**	**0.044±0.038**
**Liver**	**0.117±0.025**	0.099±0.015***	0.136±0.021***	0.086±0.013***	0.135±0.021***	0.101±0.023*
**Lungs**	**0.058±0.030**	**0.062±0.038**	0.071±0.038***	**0.049±0.022**	0.072±0.032**	**0.057±0.030**
**Kidneys**	**0.033±0.009**	**0.032±0.011**	0.041±0.012***	0.025±0.008***	0.040±0.013***	0.028±0.033*

Data are mean ± standard deviation for each study

*Significant at 10% level

**Significant at 5% level

***Significant at 1% level

When considering using 18 min of data (clinical situation) to estimate the dose, the average differences of residence time of spline method and estimation using only 18 min of data of the brain, heart, kidney, liver, and lungs are 8.38%, 15.13%, 25.02%, 23.94%, and 16.50%. However, using the 6 × 180 s frames of data required a manual filtering to make sure data with visual error was excluded, and it renders this method practically challenging.

### Comparison of bladder wall dose

**Fig. 3:**
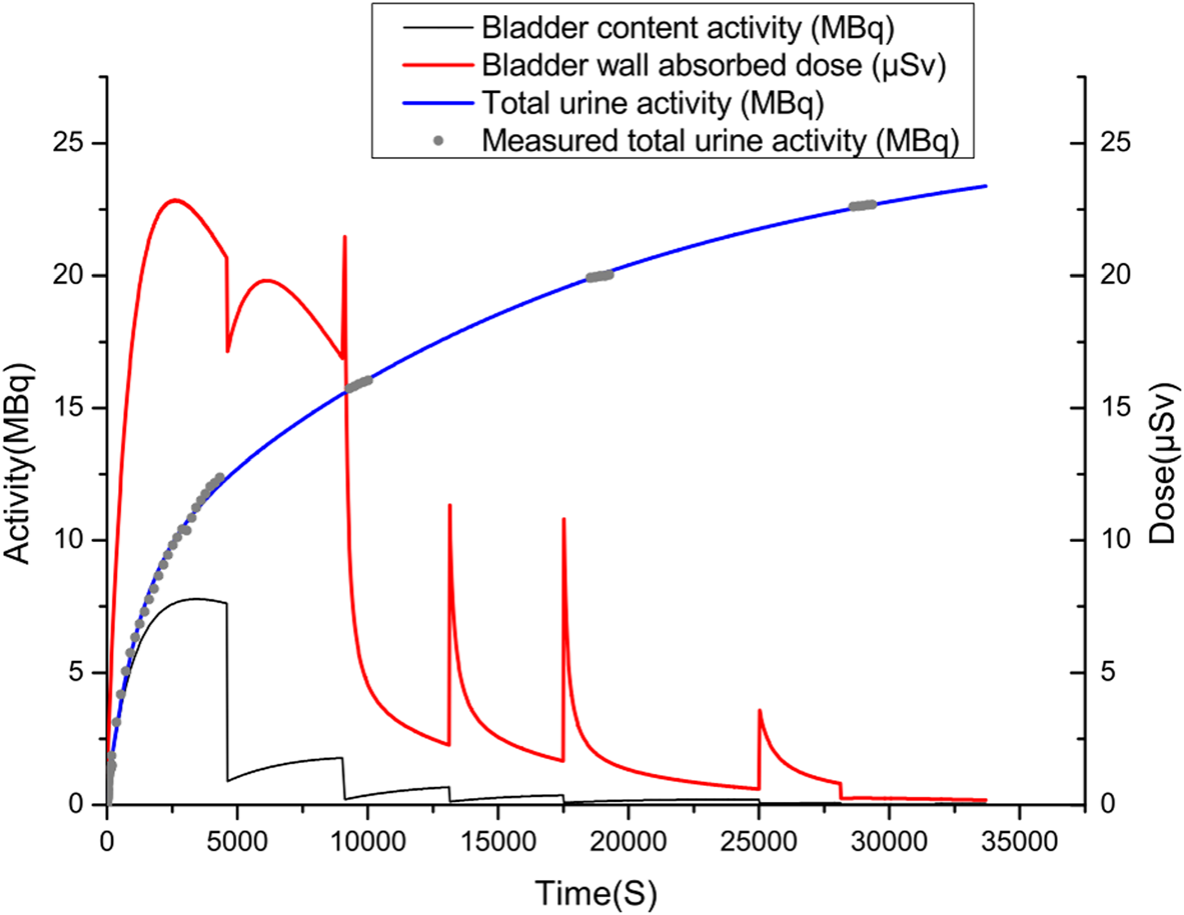
Representative time activity curve of bladder content (not decay corrected), fitting total urine activity and measured total urine activity (including bladder urine and excreted urine, decay corrected) and absorbed dose to the bladder wall

The absorbed dose of the bladder wall can be divided into two parts: the dose caused by the bladder content and the dose caused by other organs. The absorbed dose of the bladder wall caused by the bladder content can be calculated using the dynamic model which is a function of volume and activity of the bladder content (Fig. 3). The spikes of absorbed dose were caused by a rapid decrease in bladder volume after excretion, which caused a rapid increase in specific surface area of bladder content. To clarify the differences caused by long-time measurement and short-time measurement of bladder wall dose, we compared 8 h dynamic model and a dynamic model only considering two voiding. Dynamic model considering voiding within 8 h after the injection have the exact same parameters and assumptions as previous dynamic models except for the duration of measurement [17, 18].

Bladder wall dose caused by bladder content per activity of dynamic model using 8 h model and the two-voiding dynamic model are 58.63**±**23.95 and 52.69**±**23.56 μGy/MBq respectively. Bladder wall dose from 8-h dynamic model was significantly larger than that of two voiding by 13.79% (p = 0.0005 for paired t test). The assumption that the absorbed dose after the first two voiding was zero could underestimate 1.6 to 28.2% of absorbed dose. Based on the dynamic model, it is evident that the absorbed dose to the bladder per injected activity is related to the initial volume of bladder content at the beginning of the intravenous injection, urine production rate, and the time of first urine excretion.

Multivariate regression shows that all these factors are significantly related to the urine bladder wall absorbed dose per injection activity (Table 2) considering both dynamic models.

**Table 2:** Urinary bladder wall absorbed dose per injected activity multivariate regression

ln(B)	Coef. (8 h)	Standard error	P > |***t***|	Coef. (two voiding)	Standard error	P > |***t***|
**ln(F)**	**0.903**	**0.017**	**0.000**	**0.871**	**0.020**	**0.000**
**ln(I)**	**−0.055**	**0.013**	**0.004**	**−0.061**	**0.015**	**0.006**
**ln(U)**	**−0.752**	**0.195**	**0.002**	**−0.730**	**0.190**	**0.006**

B is the bladder wall absorbed dose per injected activity (μGy/MBq), F is the first voiding time (min), I is the initial volume of the bladder (mL), and U is the urine production rate (mL/min).

The result shows that urinary bladder wall absorbed dose per injected activity is positively related to the first voiding time and negatively related to the initial bladder content volume and urine production rate. The absorbed dose of bladder wall per injected activity when considering voiding in 8 h after injection can be expressed as:

10 $$ B=\frac{F^{0.903}}{I^{0.055}{U}^{0.752}} $$

The absorbed dose of bladder wall per injected activity when considering two voiding could be expressed as:

11 $$ B=\frac{F^{0.871}}{I^{0.061}{U}^{0.752}} $$

When considering different dynamic models, the correlation between the absorbed dose and urinary characteristics differ. If only two voiding is considered, the initial volume of bladder affects the bladder wall dose more than 8 h dynamic method, with the first voiding time on the contrary.

### Comparison of absorbed dose and effective dose

The absorbed dose of most target organs except the bladder wall was calculated only using S values obtained from Monte-Carlo simulation. A series of S value for male and female Asian-based revised phantoms along with the absorbed dose of each organ are presented in Additional file 1. The tissue weighting factor was based on ICRP 103 publication [30]. The absorbed dose of the bladder wall caused by other organs was calculated using S values obtained from Monte-Carlo simulation. Among all the organs, the bladder wall and the brain received the highest absorbed dose of 1.24E−01 mGy/MBq and 6.82E−02 mGy/MBq respectively, which are consistent with previous studies.

The effective dose for all subjects was 1.41E−02 ± 1.81E−03 mSv/MBq using spline method and 8 h dynamic bladder model.

When using different resident time estimation methods with different measurement of TACs, the effective dose could vary significantly. It makes appropriate assumption of the tendency of activity rather than enough data that affect the result of effective dose estimation significantly. A short-time measurement results in a significantly different estimation of effective dose with the assumption of only physical decay matters after last scan of injection by previous studies [4, 7] (Table 3). The maximum differences between long-time-measured spline fitted method and multi-exponential methods with shorter series of data was 9.93% on average.

**Table 3:** Effective dose (mSv) estimated using different residence time calculated using different methods

Spline method	Multi-exponential method 12 frames	Multi-exponential method 12 frames (only physical decay after 60 min)	Multi-exponential method 3 frames	Multi-exponential method 3 frames (only physical decay after 70 min)	6*180 s from 57 to 75 min
**1.41E−02 ± 1.81E−03**	**1.41E−02 ± 1.81E-03**	1.28E−02 ± 2.65E−03***	**1.35E−02 ± 2.72E−03**	1.27E−02 ± 2.64E−03***	1.27E−02 ± 2.66E−03***

*Significant at 10% level

**Significant at 5% level

***Significant at 1% level

Among all the investigated organs, the bladder content contributed the most to the effective dose. When using different types of dynamic bladder model, the bladder content contributed differently to the effective dose (Table 4). However, the difference of effective dose was only 1.7% on average, which is much smaller than deviation introduced by individual differences and measurement error. According to previous studies, the differences of absorbed dose estimation between MIRD and dynamic method contributed 2% differences to effective dose on average [18], which is slightly larger than the difference introduced by different dynamic models. A longer bladder dynamic model, compared with two voiding measurement, did not make more differences than the estimation model.

**Table 4:** Contribution to effective dose from different source organs

Source organs	Remainder of the body	Bladder content	Brain	Heart	Kidneys	Liver	Lungs	Effective dose (mSv)
**Attribution to effective dose (8 h)**	**38.33%**	**21.18%**	**10.86%**	**4.50%**	**3.03%**	**11.67%**	**10.43%**	**1.41E−02 ± 1.81E−03**
**Attribution to effective dose (2 voiding)**	**38.98%**	**19.80%**	**11.05%**	**4.58%**	**3.08%**	**11.88%**	**10.64%**	**1.39E−02 ± 1.81E−03**

Table 5 shows that a 50.19% difference in effective dose was caused by the difference bladder wall dose on average. It is obvious that the dynamic method helped to make an estimation much smaller than previous studies. A shorter residence time along with a dynamic model contributed to this difference. It is obvious that when using a dynamic long-time-measured TACs, considering more times of voiding helped to make a lower estimated absorbed dose of bladder. A more accurate measurement of cumulated activity is more important than dose estimation model. The bladder wall dose was at least 51.17%, lower than previous study using fixed bladder content volume (Table 5).

**Table 5:** Comparison of bladder wall dose and effective dose

	This work	Deloar et al. [4]	Mejia et al. [3]	ICRP 106 [9]
**Bladder wall dose (mGy/MBq)**	**5.86E−02 ± 2.39E−02**	**3.1E−01 ± 1.8E−01**	**1.2E−01**	**1.3E−01**
**Effective dose (mSv/MBq)**	**1.41E−02 ± 1.81E−03**	**2.9E−02 ± 9.2E−03**	**2.4E−02**	**1.9E−02**

## Discussion

To our knowledge, dosimetry studies of FDG-PET either performed a single PET procedure for less than 80 min [2–4] or performed multiple PET scans over a series of delayed intervals [7]. Even considering the organs where the cumulated activity, estimation methods have a significance impact; however, it makes no significant differences in effective dose estimation if with appropriate assumption. A short-time measurement can still make an accurate estimation if there is sufficient data and when using appropriate assumptions, even considering only 18 min of clinical data. When using a multi-exponential fitting method, a more detailed TAC does not always give a better estimation when estimating residence time. For most cases, multi-exponential fitting with the whole temporal data was unable to describe the rapid changing curve in the first minutes after injection. Furthermore, the changes of pharmacokinetics in individual within 8 h caused by urine excretion and physical activity could further make it hard to apply multi-exponential method on a long time TAC.

Besides the differences brought by different methods and measurement time, the residence time could vary significantly between individuals. The estimated residence time in the brain was significantly higher in this 6 work than values reported in previous studies [3–6, 8].

This difference could be due to the higher sensitivity of the total-body PET scanner, because of a long axial field-of-view (AFOV) of 194 cm the uEXPLORER provides a much high sensitivity of 174 kcps/MBq [31]. In comparison, due to short AFOV, conventional PET/CT scanners only have a sensitivity of less than 20 kcps/MBq [32]. uEXPLORER could provide a total-body imaging with gains of up to 40-fold compared with a conventional scanner and approximately 4-fold for single-organ imaging [33]. The higher sensitivity of the total-body PET scanner provides more accurate VOI activity measurement with smaller standard deviation, and further research will be done to investigate the higher residence time. Moreover, because of the higher sensitivity, it is more accurate to use the total-body PET to determine the radiation dose of PET tracers with shorter radioactive half-lives (such as carbon-11, nitrogen-13, and oxygen-15). Traditional scanners face the challenge of tracking the rapidly changing regional distribution of the tracer within the time course of their depositing the radiation dose to the human tissue. Total-body PET is able to record the steep time activity curve and the exact peak time of the curve to provide higher accuracy when determining the radiation dosimetry.

For all other organs except the bladder, the cumulated activity was between the reported maximum level and minimum values. Significant divergences in bladder residence time are seen in Table 6, which we believe is due to different voiding models. Compared with previous studies, a long-time measurement did not result in a significant difference for residence times of the kidneys, lung, liver, and heart.

**Table 6:** Residence time (standardized cumulated activity), in hours, used for absorbed dose estimation

Data source							
Organ	This work	Deloar et al. [4]	Hays and Segall [5]	Kaushik et al. [8]	Mejia et al. [3]	Niven at al. [6]	ICRP 106 [9]
**Brain**	**0.391±0.066**	**0.230±0.040**	**0.245±0.090**	**0.193±0.040**	**0.178±0.041**	**0.243±0.029**	**0.21**
**Heart**	0.041±0.022**	0.025±0.001**	**0.133±0.065**		**0.085±0.020**		**0.11**
**Liver**	**0.117±0.025**	**0.084±0.016**	**0.161±0.057**	**0.108±0.038**	**0.112±0.029**		**0.13**
**Lungs**	**0.058±0.030**	**0.055±0.008**	**0.084±0.028**		**0.023±0.003**		**0.079**
**Kidneys**	**0.033±0.009**	**0.036±0.019**			**0.034±0.009**		
**Bladder**	**0.124±0.032**	**0.151±0.039**	*				**0.26**
**Remainder**	**1.623±0.139**	**1.975±0.097**	**1.790±0.139**				**1.7**

*0.101 ± 0.041, 0.119 ± 0.047, and 0.191 ± 0.075 for excretion at 120, 144, and 288 min, and 0.040 ± 0.017 for voiding at 30, 60, and 120, then every 120 min

**Only left ventricle

Data are mean ± standard deviation for each study

Residual time for urine that decay outside the body: 0.252 ± 0.053

Furthermore, all the subjects in this work have a normal body mass index (male, 22.81 ± 2.79; female, 23.85 ± 3.21), and we also used an Asian-based revised phantom to estimate S value and Chinese subjects to estimate the cumulated activity. Translating this result into estimating the effective dose of different populations will need further verification.

In spite of the increase in absorbed dose of bladder when using a long-time measurement, it contributed very little when estimating the effective dose, so did different bladder models. However, previous studies often overestimate the cumulated activity of urinary bladder by underestimating the activity of the urine excreted out of the body.

Multivariate regression result shows that the absorbed dose in the bladder wall was mostly related to the urine excretion time rather than initial urine volume at injection time and urine production rate. The result is consistent with the result by Wu et al [18]. However, translating this result into estimating the absorbed dose when the voiding time is shorter than 75 min could be inaccurate, especially about 50 min after injection [18].

## Conclusion

Using a total-body PET/CT scanner, we measured time-activity curves simultaneously in all organs of the body over the time course of 8 h post-injection, and compared the accuracy of organ cumulated activity and organ dose for different methods and for different time periods of imaging. A short-time measurement can make a significant different estimation of effective dose with specific assumption. However, using only 18 min of data at 1 h after injection could make an estimation of effective dose 9.9% smaller than the result of a long-time estimation on average, which indicates that the data of clinical procedure could be used for large scale retrospective dose survey. In spite of a significant increase in bladder wall dose, a long-time dynamic bladder wall dose model makes little difference in effective dose estimation. However, when considering the absorbed dose of the bladder wall, a long-time dynamic model could give a significant different dose estimation compared with a short-time model.

## Supplementary Information

**Additional file 1.** Supplemental Data. Bladder wall dose caused by bladder content per activity of dynamic model using different methods and other urinary characteristics. Appendix A. Appendix B

## Data Availability

The data used in this study are available from the corresponding author on reasonable request.

## Abbreviations

FOV: Field of view
MIRD: Medical Internal Radiation Dose
PET: Positron emission tomography
TAC: Time-activity curve
VOI: Volume of interest
F-18 FDG: 2-[F-18]Fluoro-2-deoxy-D-glucose
