# Supplementary material for: Internal dosimetry in F-18 FDG PET examinations based on long-time-measured organ activities using total-body PET/CT: does it make any difference from a short-time measurement?
Source: EJNMMI Phys. 2021 Jul 15;8:51. doi: 10.1186/s40658-021-00395-2 (PMC8282883; doi:10.1186/s40658-021-00395-2)
Supplement: Supplementary file 1 — Additional file 1. Supplemental Data. Bladder wall dose caused by bladder content per activity of dynamic model using different methods and other urinary characteristics. Appendix A. Appendix B [file 40658_2021_395_MOESM1_ESM.docx]

**Supplemental Data**

**bladder dose caused by bladder content per activity of dynamic model using different methods and other urinary characteristics.**

| **Bladder dose per activity (8 hours)**  **(μGy/MBq)** | **Bladder dose per activity (two voiding)**  **(μGy/MBq)** | **First voiding time**  **(minute)** | **Initial bladder volume(mL)** | **Urine production rate (mL/min)** |
| --- | --- | --- | --- | --- |
| **77.42** | **65.11** | **87** | **70.00** | **0.632** |
| **51.27** | **41.98** | **85** | **63.64** | **0.839** |
| **81.91** | **80.99** | **94** | **0.00** | **1.064** |
| **60.60** | **54.77** | **87** | **0.00** | **1.782** |
| **46.44** | **36.84** | **85** | **0.00** | **2.294** |
| **23.86** | **21.00** | **88** | **63.18** | **1.941** |
| **106.85** | **103.02** | **88** | **0.00** | **1.022** |
| **50.92** | **50.14** | **128** | **0.00** | **3.125** |
| **45.54** | **40.71** | **83** | **117.55** | **1.354** |
| **41.48** | **32.34** | **77** | **240.00** | **0.519** |

**Appendix A**

|  | Male S-value (mGy/(MBq*h)) | | | | | | | **Absorbed dose** (mGy/MBq) |
| --- | --- | --- | --- | --- | --- | --- | --- | --- |
| Target organ | **Remainder of the body** | **Bladder content** | **Brain** | **Heart*** | **Kidneys** | **Liver** | **Lungs** |  |
| Skin | **2.71E-03** | **1.79E-03** | **2.14E-03** | **1.79E-03** | **2.05E-03** | **1.98E-03** | **2.03E-03** | **5.95E-03±2.09E-04** |
| Adipose tissue | **4.12E-03** | **7.27E-03** | **1.66E-03** | **4.22E-03** | **6.54E-03** | **4.09E-03** | **4.09E-03** | **9.35E-03±2.41E-04** |
| Muscle | **1.00E-02** | **3.45E-03** | **6.95E-04** | **1.85E-03** | **2.65E-03** | **1.91E-03** | **2.50E-03** | **1.76E-02±1.22E-03** |
| Adrenals | **2.15E-03** | **5.60E-04** | **1.07E-04** | **6.94E-03** | **7.03E-02** | **2.00E-02** | **5.47E-03** | **8.81E-03±7.74E-04** |
| Bladder wall | **3.72E-03** | **1.87E-01△** | **2.56E-06** | **2.17E-04** | **1.13E-03** | **5.80E-04** | **1.89E-04** | **6.81E-01±2.79E-02** |
| Blood vessel | **9.46E-03** | **1.37E-02** | **7.36E-04** | **4.59E-02** | **2.91E-02** | **2.31E-02** | **2.78E-02** | **2.49E-02±1.37E-03** |
| Brain | **5.65E-04** | **3.71E-06** | **1.66E-01** | **2.93E-04** | **6.43E-05** | **1.46E-04** | **4.51E-04** | **6.21E-02±1.13E-02** |
| Breasts | **1.45E-03** | **1.66E-04** | **3.21E-04** | **9.93E-03** | **2.09E-03** | **7.02E-03** | **8.41E-03** | **4.41E-03±4.30E-04** |
| Bronchi | **5.13E-03** | **3.82E-04** | **1.24E-03** | **9.02E-02** | **6.80E-03** | **1.83E-02** | **1.08E-01** | **2.26E-02±4.02E-03** |
| Esophagus | **1.95E-03** | **1.22E-04** | **9.67E-04** | **3.73E-02** | **3.75E-03** | **8.40E-03** | **1.57E-02** | **7.42E-03±1.07E-03** |
| Eye balls | **6.98E-04** | **6.70E-06** | **1.58E-02** | **4.74E-04** | **1.01E-04** | **2.29E-04** | **5.57E-04** | **7.06E-03±1.03E-03** |
| Eye lens | **6.58E-04** | **1.05E-15** | **1.11E-02** | **4.13E-04** | **1.29E-05** | **6.12E-05** | **5.22E-04** | **5.23E-03±7.12E-04** |
| Gall Bladder wall | **2.64E-03** | **1.44E-03** | **1.95E-04** | **1.06E-02** | **2.81E-02** | **8.35E-02** | **7.85E-03** | **1.62E-02±2.50E-03** |
| Heart wall | **1.40E-03** | **1.67E-04** | **2.73E-04** | **4.62E-01** | **3.31E-03** | **8.38E-03** | **1.74E-02** | **2.61E-02±1.09E-02** |
| Kidney | **2.24E-03** | **9.65E-04** | **6.94E-05** | **3.69E-03** | **5.94E-01** | **1.22E-02** | **2.91E-03** | **2.39E-02±6.10E-03** |
| Larynx | **1.83E-03** | **8.28E-06** | **1.88E-02** | **1.29E-03** | **1.73E-04** | **4.46E-04** | **1.71E-03** | **1.02E-02±1.16E-03** |
| LI wall | **2.55E-03** | **9.95E-03** | **4.40E-05** | **2.49E-03** | **1.06E-02** | **6.89E-03** | **1.74E-03** | **6.72E-03±2.49E-04** |
| Liver | **1.87E-03** | **5.54E-04** | **1.70E-04** | **1.09E-02** | **1.44E-02** | **1.53E-01** | **9.51E-03** | **2.27E-02±4.61E-03** |
| Lung | **2.13E-03** | **1.38E-04** | **4.64E-04** | **1.98E-02** | **3.05E-03** | **8.41E-03** | **1.52E-01** | **1.59E-02±4.89E-03** |
| Lymph node | **1.61E-03** | **5.92E-03** | **4.04E-04** | **7.56E-03** | **3.46E-03** | **3.20E-03** | **4.55E-03** | **4.62E-03±1.87E-04** |
| Oral cavity layer | **5.83E-03** | **4.72E-05** | **3.44E-02** | **3.90E-03** | **5.62E-04** | **1.74E-03** | **4.52E-03** | **2.29E-02±1.99E-03** |
| Pancreas | **1.65E-03** | **1.10E-03** | **7.79E-05** | **7.32E-03** | **1.93E-02** | **2.30E-02** | **4.01E-03** | **6.75E-03±7.03E-04** |
| Pituitary Gland | **1.14E-03** | **2.01E-15** | **6.54E-02** | **6.19E-04** | **3.34E-05** | **3.39E-05** | **7.36E-04** | **2.60E-02±4.36E-03** |
| Salivary Gland | **1.83E-03** | **1.03E-05** | **9.65E-03** | **1.05E-03** | **1.94E-04** | **4.42E-04** | **1.55E-03** | **6.76E-03±5.49E-04** |
| SI wall | **1.68E-03** | **8.35E-03** | **2.17E-05** | **1.48E-03** | **7.36E-03** | **3.08E-03** | **9.46E-04** | **4.47E-03±1.82E-04** |
| Bone surface | **3.24E-03** | **3.62E-03** | **5.62E-03** | **2.98E-03** | **2.66E-03** | **2.38E-03** | **4.29E-03** | **8.58E-03±2.33E-04** |
| Bone marrow | **7.75E-03** | **1.15E-02** | **8.75E-03** | **7.42E-03** | **7.60E-03** | **6.52E-03** | **8.94E-03** | **1.92E-02±3.79E-04** |
| Bone cartilage | **6.06E-04** | **1.45E-03** | **1.46E-04** | **5.71E-04** | **7.89E-04** | **4.53E-04** | **4.79E-04** | **1.35E-03±3.94E-05** |
| Spinal cord | **3.20E-03** | **2.83E-04** | **4.52E-03** | **1.02E-02** | **7.92E-03** | **6.76E-03** | **1.16E-02** | **9.21E-03±3.90E-04** |
| Spleen | **2.28E-03** | **3.79E-04** | **1.69E-04** | **1.13E-02** | **1.96E-02** | **4.14E-03** | **1.17E-02** | **6.23E-03±3.47E-04** |
| Stomach wall | **1.74E-03** | **6.06E-04** | **1.12E-04** | **2.03E-02** | **1.01E-02** | **1.46E-02** | **7.41E-03** | **6.40E-03±7.28E-04** |
| Thymus | **1.60E-03** | **2.98E-05** | **7.42E-04** | **1.90E-02** | **7.85E-04** | **2.38E-03** | **1.47E-02** | **5.05E-03±6.21E-04** |
| Thyroid | **3.13E-03** | **4.81E-05** | **2.17E-03** | **8.16E-03** | **6.97E-04** | **1.69E-03** | **1.11E-02** | **7.25E-03±3.87E-04** |
| Tongue | **1.24E-03** | **1.06E-05** | **5.90E-03** | **8.04E-04** | **1.09E-04** | **3.30E-04** | **1.02E-03** | **4.35E-03±3.32E-04** |
| Tonsil | **1.74E-03** | **1.50E-06** | **2.24E-02** | **5.62E-04** | **1.06E-04** | **2.31E-04** | **6.90E-04** | **1.12E-02±1.41E-03** |
| Trachea | **2.47E-03** | **2.88E-05** | **1.68E-03** | **1.52E-02** | **1.21E-03** | **2.44E-03** | **1.63E-02** | **6.78E-03±5.75E-04** |
| Ureter | **3.29E-03** | **1.95E-02** | **2.46E-05** | **1.92E-03** | **2.09E-02** | **4.77E-03** | **1.27E-03** | **9.09E-03±4.73E-04** |
| Testis | **2.65E-03** | **4.00E-03** | **7.31E-07** | **1.95E-05** | **9.26E-05** | **6.45E-05** | **1.25E-05** | **4.81E-03±2.99E-04** |
| Prostate | **5.90E-03** | **1.07E-01** | **2.06E-06** | **1.38E-04** | **8.36E-04** | **3.85E-04** | **1.11E-04** | **2.24E-02±2.53E-03** |
| *left ventricle only **△**the value is not used in the calculation, but just for reference. | | | | | | | | |
|  | | | | | | | |  |

**Appendix B**

|  | Female S-value (mGy/(MBq*h)) | | | | | | | Absorbed dose (mGy/MBq) |
| --- | --- | --- | --- | --- | --- | --- | --- | --- |
| Target organ | **Remainder of the body** | **Bladder content** | **Brain** | **Heart*** | **Kidneys** | **Liver** | **Lungs** |  |
| Skin | **2.90E-03** | **2.35E-03** | **1.96E-03** | **2.31E-03** | **2.25E-03** | **2.42E-03** | **2.62E-03** | **6.36E-03±1.08E-04** |
| Adipose tissue | **4.96E-03** | **5.59E-03** | **1.63E-03** | **3.25E-03** | **5.05E-03** | **3.59E-03** | **3.69E-03** | **1.03E-02±1.43E-04** |
| Muscle | **1.18E-02** | **3.07E-03** | **6.10E-04** | **1.86E-03** | **2.80E-03** | **1.84E-03** | **2.45E-03** | **2.01E-02±6.62E-04** |
| Adrenals | **2.20E-03** | **5.40E-04** | **1.50E-04** | **9.03E-03** | **4.62E-02** | **2.55E-02** | **6.17E-03** | **8.89E-03±2.75E-04** |
| Bladder wall | **5.18E-03** | **2.14E-01△** | **9.16E-06** | **2.61E-04** | **1.75E-03** | **7.31E-04** | **2.26E-04** | **5.98E-02±9.81E-03** |
| Blood vessel | **1.96E-02** | **3.34E-02** | **4.13E-03** | **7.64E-02** | **3.24E-02** | **3.13E-02** | **5.18E-02** | **4.69E-02±4.04E-04** |
| Brain | **6.95E-04** | **7.62E-06** | **1.83E-01** | **5.68E-04** | **1.05E-04** | **2.29E-04** | **7.66E-04** | **8.25E-02±1.29E-03** |
| Breasts | **2.54E-03** | **2.32E-04** | **1.38E-03** | **2.50E-02** | **3.31E-03** | **9.90E-03** | **1.81E-02** | **7.42E-03±1.37E-04** |
| Bronchi | **1.27E-03** | **6.58E-05** | **3.56E-04** | **2.99E-02** | **1.51E-03** | **3.57E-03** | **2.32E-02** | **4.45E-03±1.67E-04** |
| Esophagus | **3.22E-03** | **1.54E-04** | **1.84E-03** | **4.70E-02** | **4.72E-03** | **1.15E-02** | **2.33E-02** | **9.79E-03±3.39E-04** |
| Eye balls | **1.33E-03** | **4.27E-05** | **2.73E-02** | **1.32E-03** | **2.86E-04** | **6.33E-04** | **1.47E-03** | **1.44E-02±2.33E-04** |
| Eye lens | **8.83E-04** | **3.62E-06** | **2.15E-02** | **2.09E-03** | **8.01E-05** | **5.33E-04** | **1.47E-03** | **1.11E-02±1.72E-04** |
| Gall Bladder wall | **3.95E-03** | **1.53E-03** | **2.27E-04** | **1.41E-02** | **5.80E-02** | **8.87E-02** | **9.21E-03** | **2.00E-02±1.20E-03** |
| Heart wall | **1.62E-03** | **1.60E-04** | **4.64E-04** | **5.45E-01** | **3.67E-03** | **9.28E-03** | **2.08E-02** | **2.08E-02±6.80E-03** |
| Kidney | **3.12E-03** | **1.31E-03** | **1.05E-04** | **4.44E-03** | **6.79E-01** | **1.70E-02** | **3.58E-03** | **3.29E-02±1.60E-03** |
| Larynx | **1.61E-03** | **1.56E-05** | **1.39E-02** | **1.68E-03** | **1.97E-04** | **4.62E-04** | **2.10E-03** | **8.92E-03±1.62E-04** |
| LI wall | **4.87E-03** | **2.01E-02** | **3.96E-05** | **1.15E-03** | **1.12E-02** | **4.53E-03** | **9.00E-04** | **1.15E-02±6.44E-04** |
| Liver | **2.17E-03** | **6.47E-04** | **2.49E-04** | **1.19E-02** | **1.76E-02** | **1.70E-01** | **1.02E-02** | **2.49E-02±2.54E-03** |
| Lung | **2.85E-03** | **1.70E-04** | **8.03E-04** | **2.64E-02** | **3.76E-03** | **1.04E-02** | **2.00E-01** | **1.48E-02±1.47E-03** |
| Lymph node | **1.57E-03** | **3.84E-03** | **5.23E-04** | **5.11E-03** | **4.84E-03** | **2.85E-03** | **3.68E-03** | **4.07E-03±9.20E-05** |
| Oral cavity layer | **5.39E-03** | **5.18E-05** | **3.07E-02** | **6.26E-03** | **9.09E-04** | **2.05E-03** | **7.09E-03** | **2.30E-02±4.61E-04** |
| Pancreas | **2.58E-03** | **1.70E-03** | **1.31E-04** | **6.13E-03** | **4.01E-02** | **2.35E-02** | **3.83E-03** | **9.00E-03±2.86E-04** |
| Pituitary Gland | **1.60E-03** | **1.49E-15** | **7.44E-02** | **9.87E-04** | **1.63E-04** | **3.65E-04** | **9.73E-04** | **3.57E-02±5.59E-04** |
| Salivary Gland | **2.27E-03** | **2.57E-05** | **1.62E-02** | **2.22E-03** | **3.28E-04** | **7.76E-04** | **2.96E-03** | **1.11E-02±2.11E-04** |
| SI wall | **2.77E-03** | **1.70E-02** | **4.29E-05** | **1.70E-03** | **1.34E-02** | **5.40E-03** | **1.13E-03** | **7.97E-03±6.45E-04** |
| Bone surface | **4.22E-03** | **3.36E-03** | **6.35E-03** | **3.13E-03** | **3.30E-03** | **2.72E-03** | **4.45E-03** | **1.07E-02±1.77E-04** |
| Bone marrow | **8.82E-03** | **1.03E-02** | **1.19E-02** | **9.19E-03** | **9.61E-03** | **8.02E-03** | **1.05E-02** | **2.28E-02±3.17E-04** |
| Bone cartilage | **4.47E-03** | **4.90E-03** | **7.81E-04** | **5.55E-03** | **5.41E-03** | **5.66E-03** | **4.32E-03** | **9.35E-03±1.30E-04** |
| Spinal cord | **2.21E-03** | **1.38E-04** | **3.28E-03** | **4.92E-03** | **5.00E-03** | **4.20E-03** | **6.45E-03** | **6.08E-03±1.27E-04** |
| Spleen | **2.73E-03** | **5.16E-04** | **2.69E-04** | **1.37E-02** | **2.72E-02** | **7.42E-03** | **1.26E-02** | **7.34E-03±6.79E-05** |
| Stomach wall | **2.66E-03** | **1.15E-03** | **2.25E-04** | **1.91E-02** | **2.38E-02** | **2.20E-02** | **8.80E-03** | **8.88E-03±1.40E-04** |
| Thymus | **1.88E-03** | **4.85E-05** | **1.07E-03** | **2.09E-02** | **9.12E-04** | **2.46E-03** | **1.51E-02** | **5.01E-03±1.72E-04** |
| Thyroid | **3.77E-03** | **4.51E-05** | **3.79E-03** | **9.24E-03** | **9.00E-04** | **2.07E-03** | **1.16E-02** | **8.71E-03±2.38E-04** |
| Tongue | **1.54E-03** | **1.76E-05** | **1.03E-02** | **2.04E-03** | **2.89E-04** | **7.18E-04** | **2.33E-03** | **7.26E-03±1.39E-04** |
| Tonsil | **2.00E-03** | **2.51E-05** | **1.49E-02** | **1.45E-03** | **2.38E-04** | **4.42E-04** | **2.06E-03** | **9.99E-03±1.90E-04** |
| Trachea | **4.19E-03** | **9.50E-05** | **3.42E-03** | **2.52E-02** | **1.44E-03** | **3.58E-03** | **2.45E-02** | **1.04E-02±2.71E-04** |
| Ureter | **3.46E-03** | **1.71E-02** | **4.21E-05** | **1.51E-03** | **1.81E-02** | **5.49E-03** | **1.15E-03** | **9.27E-03±6.17E-04** |
| Ovaries | **5.21E-03** | **3.85E-02** | **2.49E-06** | **1.57E-04** | **1.39E-03** | **6.55E-04** | **1.75E-04** | **1.37E-02±1.37E-03** |
| Uterine wall | **5.12E-03** | **8.93E-02** | **1.04E-05** | **2.30E-04** | **1.69E-03** | **7.13E-04** | **2.08E-04** | **2.04E-02±3.61E-03** |
| *left ventricle only **△**the value is not used in the calculation, but just for reference. | | | | | | | |  |
